# An Investigation of the Prognostic Role of Genes Related to Lipid Metabolism in Head and Neck Squamous Cell Carcinoma

**DOI:** 10.1155/2023/9708282

**Published:** 2023-02-10

**Authors:** Ling Qian, Chenyu Zhou, Keyi Wang, Liuyang Li, Wenhui Xia, Yuan Fan

**Affiliations:** ^1^Department of Oral Mucosal Diseases, The Affiliated Stomatological Hospital of Nanjing Medical University, Nanjing, China; ^2^Jiangsu Province Key Laboratory of Oral Diseases, Nanjing Medical University, Nanjing, China; ^3^Jiangsu Province Engineering Research Center of Stomatological Translational Medicine, Nanjing, China

## Abstract

Head and neck squamous cell carcinoma (HNSCC) has become a prevalent malignancy, and its incidence and mortality rate are increasing worldwide. Accumulating evidence has indicated that lipid metabolism-related genes (LMRGs) are involved in the occurrence and development of HNSCC. This study investigated the latent association of lipid metabolism with HNSCC and established a prognostic signature based on LMRGs. A prognostic risk model composed of eight differentially expressed LMRGs (*PHYH*, *CYP4F8*, *INMT*, *ELOVL6*, *PLPP3*, *BCHE*, *TPTE*, and *STAR*) was constructed through The Cancer Genome Atlas database. Then, ELOVL6 expression was validated in oral squamous cell carcinoma (OSCC), which is a common type of HNSCC, by immunohistochemical analysis. ELOVL6 expression in the OSCC II/III group was significantly higher than that in the other three groups (normal, dysplasia, and OSCC I), and OSCC patients with high ELOVL6 expression had poorer survival than those with low ELOVL6 expression. In summary, the LMRG-based prognostic feature had prognostic predictive capacity. ELOVL6 may be a potential prognostic factor for HNSCC patients.

## 1. Introduction

According to statistics, there are approximately 600,000 new cases of head and neck cancer annually worldwide [1–4]. Head and neck squamous cell carcinoma (HNSCC), the main pathological type of head and neck cancer, is a heterogeneous group of malignancies that predominantly arise from the epithelium of the upper aerodigestive tract. Although varying combinations of surgery, chemoradiotherapy, and targeted therapy can be selected according to the staging of HNSCC, deaths from HNSCC have not declined. A total of 380,000 people die from this disease every year [1, 5], and its 5-year survival rate is approximately 60% [4, 6–10]. Moreover, HNSCC often arises from pre-existing oral diseases, such as oral lichen planus and oral leukoplakia. Liquid biopsy has shown great potential in the diagnosis and monitoring of precancerous lesions and HNSCC because of its minimally invasive and reproducible nature. Various circulating biomarkers (circulating cell-free DNA, miRNA, proteins, and exosomes) in blood and saliva can change quantitatively or even qualitatively in the early stages of the disease, leading to or promoting tumour initiation and progression. The detection and analysis of biomarkers in peripheral blood and saliva, could establish the dynamic monitoring of HNSCC in spatial and temporal aspects and improve early diagnosis and real-time monitoring of the disease in a precise and personalized manner [11–14].

In recent years, increasing evidence has shown that reprogrammed metabolism may play a critical role in the progression of tumours [9, 15]. Lipid metabolic reprogramming is one of the hallmarks of cancer and a key factor in tumour growth, metastasis, and resistance. When blood lipids cannot meet the needs of rapid tumour growth, more lipids need to be generated through *de novo* lipogenesis [9, 16]. The aim of increased lipogenesis is mainly to synthesize more cytosolic lipids to meet the needs of rapidly proliferating cancer cells and their surging energy demand. Studies have shown that activation of lipid metabolism is associated with poor prognosis in a variety of tumours, mainly due to the upregulation of lipid metabolism-related genes (LMRGs) at multiple levels, such as transcription, translation, posttranslational modification, and enzymatic activity [17–21]. Moreover, LMRGs are not only prognostic markers of tumours, but can also be used as targets for inhibiting tumour neogenesis [22]. Acetyl-CoA carboxylase 2, which is involved in cell energy and lipid metabolism, is highly expressed in laryngocarcinoma [23]. The overexpression of fatty acid synthesis is associated with poor prognosis in HNSCC [24]. However, the prognostic role of LMRGs in HNSCC remains poorly understood, and more analysis of their prognostic value and function needs to be explored.

In the present study, we used a bioinformatics method to determine the underlying mechanism associated with the differentially expressed genes (DEGs) identified by gene set enrichment analysis (GSEA) of normal and HNSCC samples and then screened the differentially expressed LMRGs and evaluated their clinical prognostic value. These signatures further confirmed that our prognostic prediction model could serve as an independent prognostic factor. Importantly, we investigated the relationship between immunity and differentially expressed LMRGs (DELMRGs). Finally, we validated ELOVL6 expression and its prognostic value in oral squamous cell carcinoma (OSCC), which is a common type of HNSCC.

## 2. Materials and Methods

### 2.1. Data Collection

The Cancer Genome Atlas (TCGA)-HNSCC database contains gene expression data and corresponding clinical data for 502 HNSCC patients and 44 normal subjects. Patients who lacked clinical data were excluded. Finally, as many as 499 HNSCC patients were identified and randomly assigned to the training set (*n* = 349) and validation set (*n* = 150) at a ratio of 7 : 3. In addition, the single nucleotide polymorphism data of 506 HNSCC patients were also obtained from the TCGA database. LMRGs were obtained from the four lipid metabolism-related datasets [Reactome metabolism of lipids and lipoproteins, Reactome phospholipid metabolism, Hallmark fatty acid metabolism, and Kyoto Encyclopedia of Genes and Genomes (KEGG) glycerophospholipid metabolism] in the Molecular Signature Database.

### 2.2. GSEA and Identification of DEGs

GSEA was conducted to determine the significantly upregulated and downregulated lipid metabolism-related pathways between normal and HNSCC samples using *P* < 0.01 as the cut-off criterion. The DEGs between normal and HNSCC samples in the TCGA database were identified using the ‘limma' R package. The |log_2_-fold change (FC)| ≥ 1 and false discovery rate (FDR) < 0.05 were regarded as the cut-off criteria. A volcano plot was created to visualize the differences in gene expression levels between the two groups. After overlapping with LMRGs, we identified the DELMRGs.

### 2.3. Establishment of the Lipid Metabolism-Related Signature

The 206 DELMRGs in the training set were subjected to univariate Cox regression analysis incorporating the Kaplan–Meier (K–M) method using the survival R package. Genes with *P* < 0.05 were then integrated into the multivariate Cox regression analysis with a step function to situate the optimal variables for constructing the prognostic feature. Risk scores were generated for each sample from the values of the coefficients and expressions of the best variables under the following formula.

(1) $$\begin{array}{c} \text{Risk score}=\beta_{1}\times {\text{Exp}}_{1}+\beta_{2}\times {\text{Exp}}_{2}+...+\beta_{i}\times {\text{Exp}}_{i}, \end{array}$$

where *β* represents the coefficient value and Exp represents the gene expression level. The ‘survminer' software package in R was used to determine the optimal threshold for risk scores to classify patients into high- and low-risk groups. The assessment of the difference in overall survival (OS) between high- and low-risk groups relied on K–M survival curves with log-rank tests. Receiver operating characteristic (ROC) curves were plotted with the ‘SurvivalROC' package, whereas the R ‘survival ROC' package calculated the area under the curve (AUC) of the ROC to evaluate the prognostic ability of the above feature. Furthermore, a scatter plot of the risk scores was generated with the ‘pheatmap' package in R. Finally, the results above were verified with the validation set. Notably, we performed K–M survival analysis on each gene selected by univariate Cox regression analysis, and the gene with the most significant survival differences between the high- and low-risk groups was used for immunohistochemical (IHC) analysis.

### 2.4. Construction of a Protein–Protein Interaction Network

The STRING platform (https://www.string-db.org/), which is a web resource that can predict gene interactions at the protein level, was used to construct protein–protein interaction (PPI) networks.

### 2.5. Analysis of the Prognostic Independence of the Gene Signature Related to Lipid Metabolism

The assessment of independent prognostic factors in patients with HNSCC was performed with univariate and multivariate Cox regression analyses. The variables included were risk score, age, sex, T, N, M, and stage. Variables with *P* < 0.05 were considered statistically significant. In addition, nomograms were drawn based on the results of the multivariate Cox regression using the ‘rms' package.

### 2.6. Functional Enrichment Analysis

DEGs meeting the criteria of |log_2_FC| ≥ 1 and FDR < 0.05 between high- and low-risk groups were labelled by the ‘edgeR' package. The primary functions of these DEGs were revealed by Gene Ontology (GO) and KEGG analyses. The GO analysis terms included cellular component (CC), molecular function (MF), and biological process (BP). *P*-values less than 0.05 were considered statistically significant.

### 2.7. Immune Cell Infiltration Analysis

The following two methods were used to determine the relationship between risk scores and the immune microenvironment. The ESTIMATE algorithm was used to analyse the stromal and immune scores of the high- and low-risk groups. Moreover, the abundance of 22 immune cell subtypes in HNSCC was derived by the CIBERSORT algorithm. A violin plot reflecting the difference in the infiltration levels of immune cells between the two risk groups was drawn using the ‘vioplot' package in the R software. The correlation between the eight immune cells and eight prognostic genes was evaluated using Spearman's correlation analysis. In addition, the threshold of Spearman's correlation coefficient was set as (|*R*| > 0.3) and *P* < 0.05.

### 2.8. Patient Samples

The research, which was approved by the Nanjing Medical University Ethics Committee, Nanjing, China, was conducted in accordance with the World Medical Association Declaration of Helsinki. All individuals who participated in the study provided informed consent under the approved guidelines set by the ethics committee at Nanjing Medical University, Nanjing, China.

Patients who had not undergone preoperative chemoradiotherapy or other immunotherapy were selected for inclusion in this study. A total of 138 archived cases from 2015 to 2017 were collected from the Department of Pathology, the Affiliated Stomatological Hospital of Nanjing Medical University, Nanjing, China. Thirty normal oral mucosa tissues, 37 dysplasia tissues, and 71 OSCC tissues were included, with 19 pairs of cancerous and paracancerous tissues. The clinicopathological characteristics were collected, including sex, age, tumour–node–metastasis (TNM) stage, pathology tumour stage, pathology node stage, preoperative metastasis, perineural/vascular invasion, tumour location, and OS. The TNM stage of OSCC was classified according to the seventh edition of the American Joint Committee on Cancer.

### 2.9. IHC Analysis

IHC was performed to evaluate ELOVL6 expression by the standard Envision method. Briefly, paraffin-embedded tissue blocks were cut into 4 *μ*m thick sections and treated with xylene. Then, each section was dewaxed and rehydrated in a graded ethanol series. Hydrogen peroxide (3%) was used to inhibit endogenous peroxidase. A 1 : 300 dilution of polyclonal rabbit antibody against ELOVL6 (ab69857; Abcam, Cambridge, MA, USA) was added and incubated overnight at 4°C. The reactions were visualized by the Envision peroxidase kit (Dako, Carpinteria, CA, USA). Horseradish peroxidase was used, followed by incubation with 3,3′-diaminobenzidine for colouration. Finally, the sections were counterstained with haematoxylin, followed by a graded ethanol series, and then sealed with xylene. The results were assessed by a semiquantitative *H*-score, which was combined with the intensity of staining (0 = no; 1 = weak; 2 = moderate; and 3 = strong) and the percentage of staining (1 = 0–25%; 2 = 26–50%; 3 = 51–75%; and 4 = 76–100%). The final IHC score was obtained by multiplying the staining score and percentage staining score, with a minimum of 0 and a maximum of 12. Based on the final results, a cut-off value of 6 was calculated by X-tile, and the patient samples were divided into a high expression group (>6) and a low expression group (≤6) according to the cut-off value. IHC results were analysed by a single independent readings by two experienced and blinded pathologists.

### 2.10. Statistical Analysis

TCGA statistical analyses were performed with the R software (Version 3.6.1). The Mann–Whitney *U* test was performed to compare the TMB of the high- and low-risk groups. Statistical analyses of IHC were conducted by the SPSS 25.0. *χ*^2^ and Fisher's precision probability tests were used to examine the relationship between ELOVL6 expression and clinicopathological parameters. The Wilcoxon signed rank test was used to evaluate the expression of ELOVL6 in paired samples. Using X-tile, cut-offs were calculated based on clinicopathological data and patient survival. Survival curves were analysed by the K–M method. *P* < 0.05 was considered statistically significant if not otherwise stated.

## 3. Results

### 3.1. Identification of LMRGs in HNSCC

**Figure 1:**
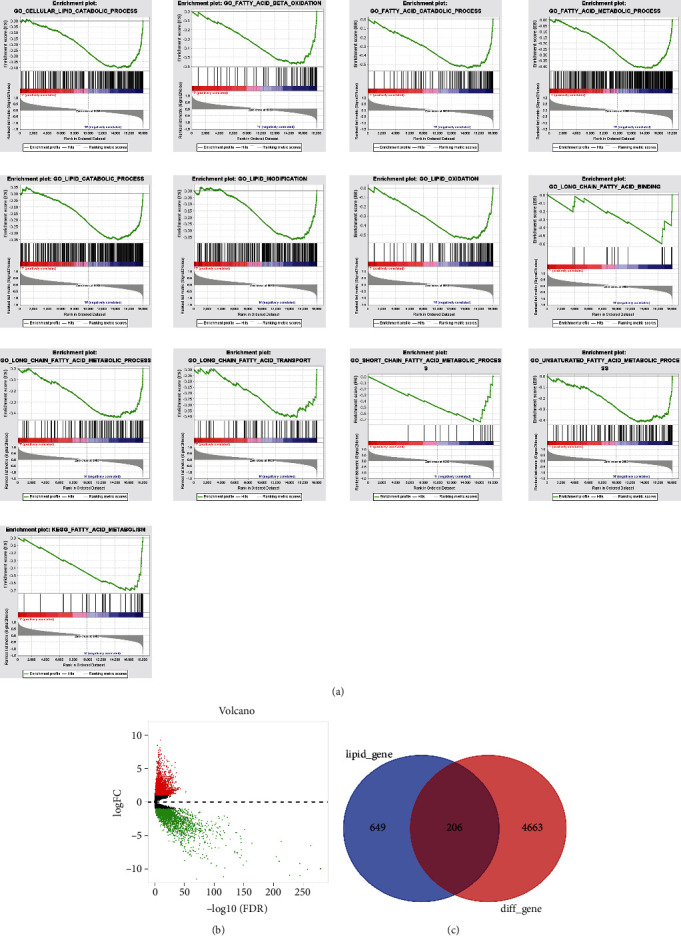
Screening of LMRGs in HNSCC. (a) GSEA enrichment analyses of lipid metabolism-related biological pathways between HNSCC and normal samples. (b) Distribution of DEGs between HNSCC and normal samples. (c) The intersection of the LMRGs and DEGs.

We analysed all genes between normal and tumour samples by GSEA and found that a variety of lipid metabolism-related biological pathways were activated, such as ‘fatty acid metabolism', ‘long-chain fatty acid binding', ‘fatty acid beta oxidation', ‘fatty acid catabolic process', ‘lipid modification', and ‘lipid oxidation'. The detailed results are shown in Figure 1(a). For this reason, we speculated that lipid metabolism may play an important role in HNSCC. Then, we first analysed the DEGs between normal and HNSCC samples, and a total of 4869 DEGs (2499 upregulated and 2370 downregulated) were identified. The volcano plot clearly presents the distribution of these DEGs (Figure 1(b)). From the intersection of the 855 LMRGs and 4869 DEGs, a total of 206 DELMRGs were identified, as shown in Figure 1(c).

### 3.2. Construction and Verification of the HNSCC Risk Model

**Figure 2:**
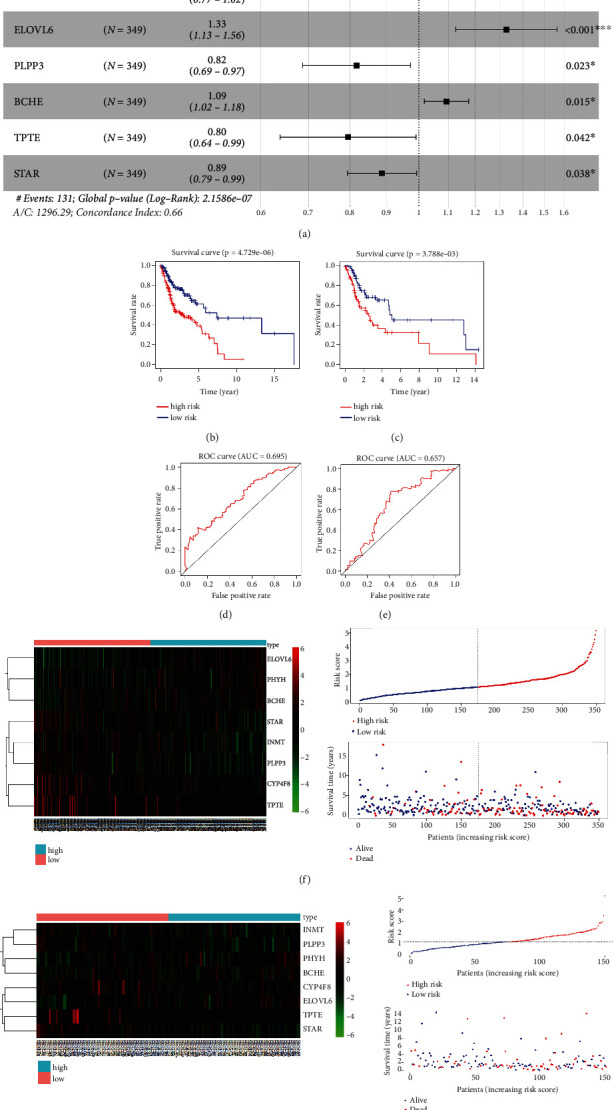
Establishment of the HNSCC risk model. (a) Construction of eight prognosis genes (*PHYH*, *CYP4F8*, *INMT*, *ELOVL6*, *PLPP3*, *BCHE*, *TPTE*, and *STAR*) by multivariate Cox. (b and c) Kaplan–Meier curves of the eight-gene signature in the training and testing set. (d and e) The AUC of the ROC curve in the training set and testing set was 0.695 and 0.657, respectively. (f) The expression patterns of eight identified DELMRGs for the patients in the training set (left). The distribution of risk score (top right) and vital status (bottom right) of patients in the training set. (g) The heatmap of mRNA expression of the eight-gene signature (left) and the distribution of risk score (right) in the testing set.

Through univariate Cox regression analysis and K–M survival analysis, 19 out of the 206 DELMRGs were identified as closely related to HNSCC patient OS in the training set (*P* < 0.05), and the K–M curve generated based on ELOVL6 expression showed the most significant survival differences between the high- and low-risk groups (*P* = 0.001; Table 1). Then, 19 DELMRGs were further subjected to multivariate Cox regression analysis, and 8 candidate DELMRGs (*PHYH*, *CYP4F8*, *INMT*, *ELOVL6*, *PLPP3*, *BCHE*, *TPTE*, and *STAR*) were identified as prognostic genes (Figure 2(a)).

**Figure 3:**
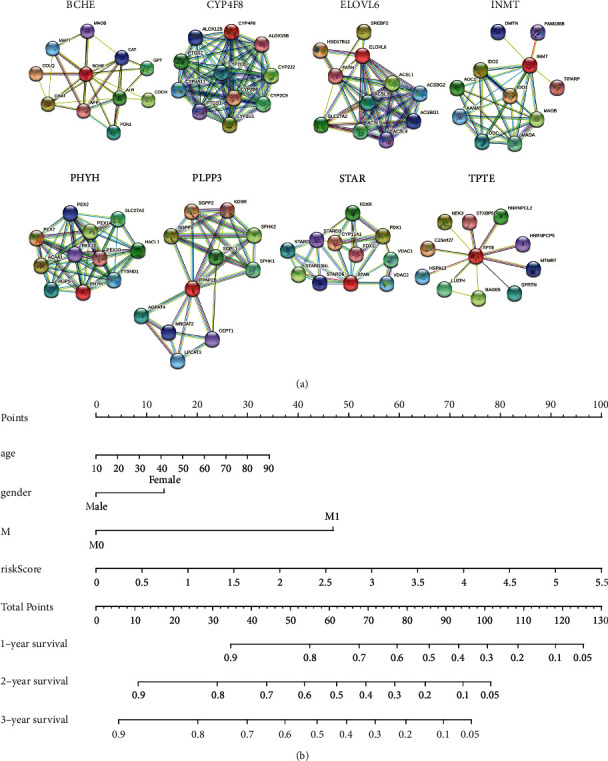
Analyses of the HNSCC risk model. (a) PPI network of eight prognostic genes. (b) Nomogram to predict 1-, 3-, or 5-year OS in the TCGA-HNSCC training set.

Patients were subsequently classified into two groups, namely, high- and low-risk groups, based on the median risk score. The K–M survival analysis suggested that patients with high-risk scores had a remarkably worse survival rate than those with low-risk scores in the training and testing sets (Figures 2(b) and 2(c)). As shown in Figures 2(d) and 2(e), the area under the ROC curve values reached 0.695 and 0.657 in the training set and testing set, respectively, exhibiting good accuracy. Furthermore, the scatterplot revealed a clustering of deaths in a high-risk trend. We described the expression patterns of prognostic genes in the two groups using heatmaps (Figures 2(f) and 2(g)). In summary, these results indicated that the risk score showed satisfactory performance in predicting the OS for HNSCC. In addition, we searched the genes that might interact with the eight prognostic signatures by constructing a PPI network (Figure 3(a)).

### 3.3. Prognostic Features Based on LMRGs Were Independent Prognostic Factors for HNSCC

To explore the independent prognostic value of the risk score, we included the risk score and clinical characteristics (gender, age, stage, T, N, and M) in univariate and multivariate Cox regression analyses. As shown in Table 2, age, sex, M stage, and risk score were considered independent prognostic factors (*P* < 0.05). In addition, we integrated the risk score and age, sex, and M stage to build a nomogram to evaluate the clinical traits and prognostic model for HNSCC patient prognosis (Figure 3(b)).

### 3.4. Functional Enrichment Analysis of Genes Associated with the Prognostic Feature

**Figure 4:**
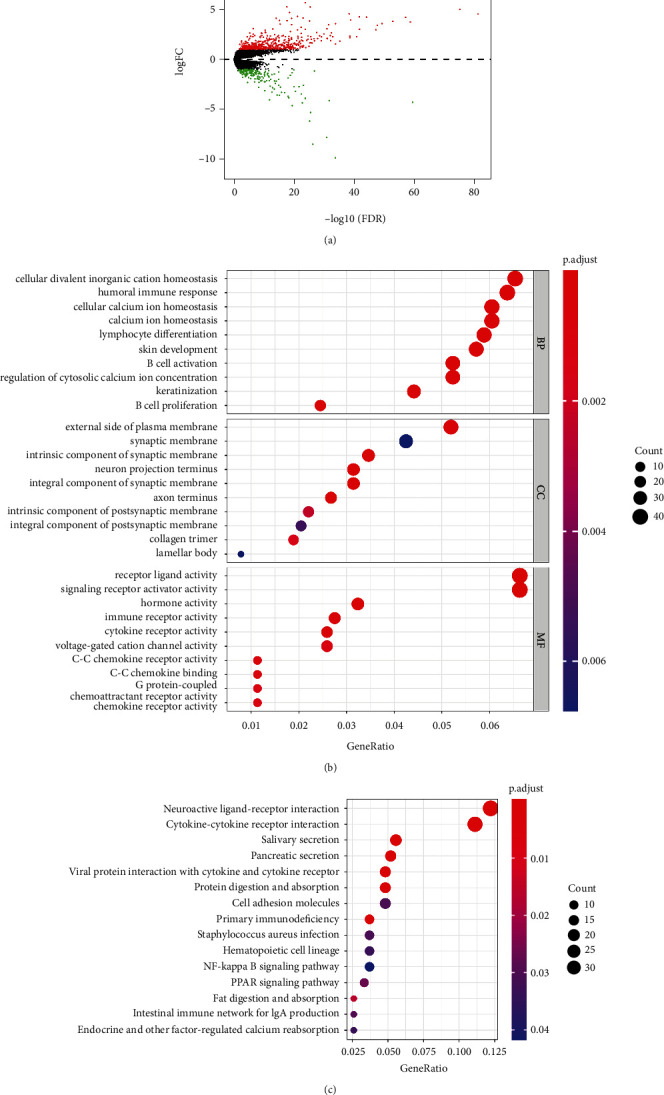
The biological functions and signalling pathways of the prognostic signature. (a) Volcano plot of the 702 DEGs between high- and low-risk groups. (b) The top 10 enriched GO BP, CC, and MF terms. (c) The most important KEGG pathways in DEGs between high- and low-risk groups.

To explore the potential signalling pathways related to the risk score in HNSCC, we screened DEGs between the high- and low-risk groups and analysed them with GO and KEGG analyses. A total of 702 DEGs were identified, including 511 upregulated genes and 191 downregulated genes (Figure 4(a)). We found that the most significant GO enriched terms were cellular divalent inorganic cation homeostasis, cellular calcium ion homeostasis (BP), external side of plasma membrane (CC), receptor ligand activity, and signalling receptor activator activity (MF), as shown in Figure 4(b). In the KEGG enrichment analysis, the DEGs were primarily correlated with the neuroactive ligand–receptor interaction, cytokine–cytokine receptor interaction, salivary secretion, pancreatic secretion, and viral protein interaction with cytokines and cytokine receptors (Figure 4(c)). Moreover, we found that DEGs were also related to some immune functions, including humoral immune response, B-cell activation, B-cell proliferation, primary immunodeficiency, and intestinal immune network for Immunoglobulin A (IgA) production.

### 3.5. The Relationship between the Risk Score and the Immune Microenvironment of HNSCC

**Figure 5:**
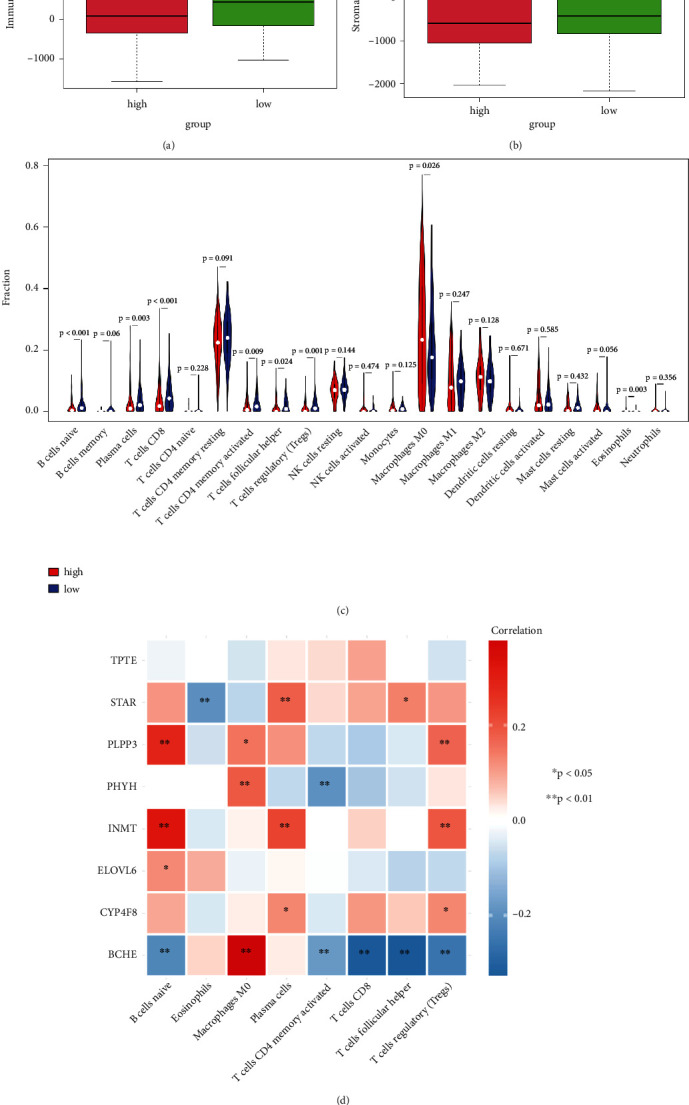
The correlation between immunity and prognostic signature. (a and b) The immune and stromal scores between high- and low-risk groups. (c) The fraction of 22 immune cells between two risk groups. (d) The correlation between eight immune cells and eight prognostic genes.

The DEGs between the high- and low-risk groups participated in a variety of immune-related pathways according to the results of functional enrichment analysis. Thus, we compared the stromal and immune scores between the two risk groups, and the low-risk group had higher stromal and immune scores than the high-risk group (Figures 5(a) and 5(b)). We further measured the relative proportions of 22 immune cells between the two risk groups. The low-risk group had increased infiltration levels of naive B cells, plasma cells, CD8 T cells, activated memory CD4 T cells, follicular helper T cells, and regulatory T cells. Conversely, it decreased the infiltration of M0 macrophages and eosinophils (Figure 5(c)). Consequently, we further analysed the correlation between eight immune cells and eight prognostic signatures. The results from Figure 5(d) indicated that a significant positive correlation was found between the expression of INMT and naive B cells. Furthermore, BCHE expression was negatively correlated with CD8 T cells and follicular helper T cells, whereas it was positively correlated with M0 macrophages.

### 3.6. ELOVL6 Expression Was Associated with Poor Prognosis according to IHC

**Figure 6:**
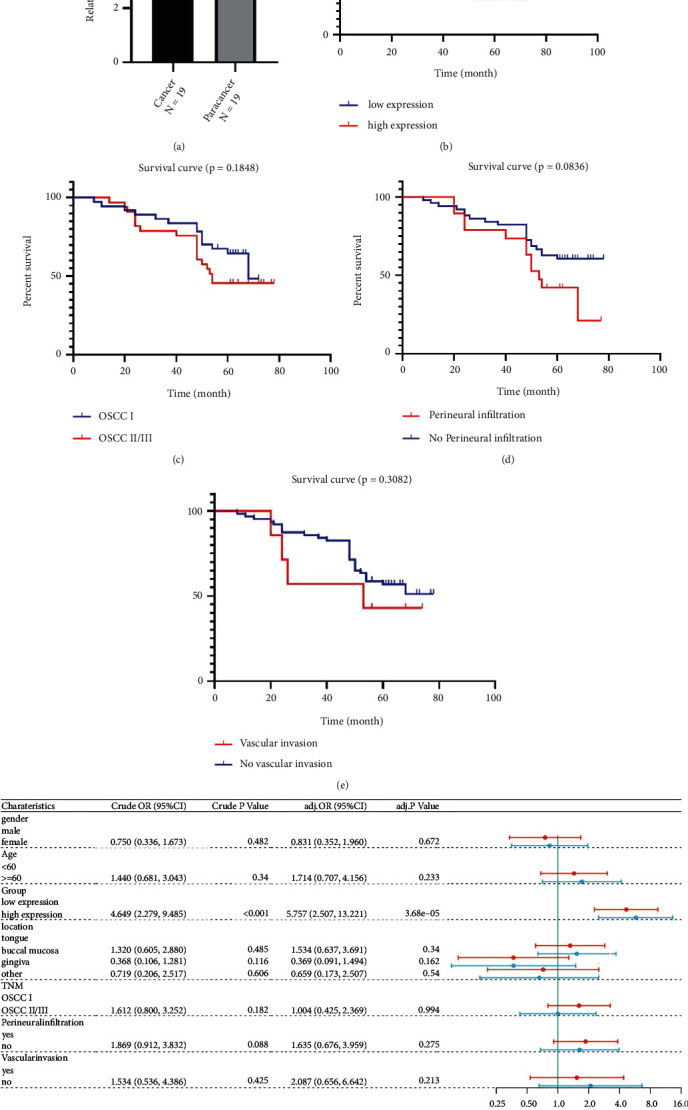
Analyses of the ELOVL6 expression by IHC. (a) ELOVL6 expression in cancer and adjacent noncancerous tissues. (b–e) Survival curves of OSCC generated by ELOVL6 expression, TNM stage, and perineural and vascular infiltration. (f) Cox regression analyses in OSCC patients through clinicopathological features.

**Figure 7:**
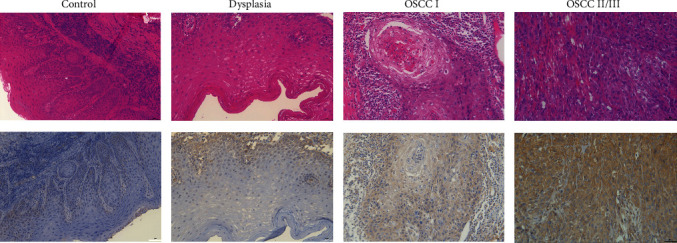
The ELOVL6 expression by IHC in normal, dysplasia, OSCC I, and OSCC II/III (×200). ELOVL6 mainly expressed in the cytoplasm and a little in the nucleus.

ELOVL6 is mainly expressed in the cytoplasm and partly in the nucleus and cytomembrane according to IHC. The results also confirmed that the relative expression level of ELOVL6 in 19 OSCC tissues was significantly higher than that in adjacent noncancerous tissues (*P* < 0.001; Figure 6(a)). All enrolled patients were divided into four groups, namely, the normal group, dysplasia group, OSCC I group, and OSCC II/III group. Moreover, the patients were divided into a high expression group (>6) and a low expression group (≤6) according to the cut-off value of 6. The rate of high ELOVL6 expression was 45.5% in the OSCC II/III group and 16.7%, 5.4%, and 18.4% in the normal, dysplasia, and OSCC I groups, respectively, and the rate of high ELOVL6 expression in the OSCC II/III group was significantly higher than that in the other three groups (Table 3; Figure 7).

Our study shows that IHC expression of ELOVL6 in 71 OSCC patients was only correlated with TNM stage, whereas no statistically significant differences were observed in patient sex, age, tumour location, tumour size, lymph node metastasis, or vascular and perineural invasion (Table 4).

K–M survival curves show that OSCC patients with high ELOVL6 expression had poorer survival than those with low ELOVL6 expression, and one patient who died from a nononcological cause was excluded. However, OS was poorly correlated with TNM stage and perineural and vascular invasion (Figures 6(b), 6(c), 6(d), and 6(e)). In addition, we confirmed that ELOVL6 expression was an independent risk factor for OSCC patient prognosis by Cox regression analyses (Figure 6(f)).

## 4. Discussion

Lipid metabolism is one of the most prominent metabolic alterations in cancer [25]. A reduction in the expression of certain metabolic genes has been reported to improve survival in patients with human papilloma virus (HPV)-positive HNSCCs [26]. Recent studies have also indicated that there may be a causal link between obesity and higher risks and mortality of head and neck cancer [27, 28], and researchers have also found that fatty acid metabolism-related enzyme expression is upregulated in patients with advanced HNSCC, which might be a poor prognosis for survival outcomes [24, 29]. Although it has been suggested that HNSCC has dysregulated lipid metabolism, the role of this dysregulated lipid metabolism has not been fully elucidated. Furthermore, bioinformatics tools have been widely used to identify novel molecular markers of cancer. In HNSCC, many scholars use bioinformatics methods to identify biomarker candidates for diagnosis and prognosis, such as the biomarkers of tumour-infiltrating lymphocytes and HPV-related tumour microenvironment remodelling [30–32]. However, the biomarkers obtained from bioinformatics represent only the starting point, further experimental and functional studies would have to be performed in order to validate their predictive role [33]. Indeed, some novel molecular markers have also been sought and clinically validated through the above approaches [34].

In the present study, we found that multiple lipid metabolism-related pathways were activated according to GSEA. On these grounds, we speculated that lipid metabolism played a crucial role in the development and progression of HNSCC. Subsequently, we identified the DELMRGs in HNSCC and further evaluated their prognostic value. The results indicated that eight LMRGs (*PHYH*, *CYP4F8*, *INMT*, *ELOVL6*, *PLPP3*, *BCHE*, *TPTE*, and *STAR*) were related to OS of HNSCC patients. Most of these genes have been shown to be involved in cancers. Among them, ELOVL6 is associated with the poor prognosis of patients with hepatocellular carcinoma, triple-negative breast cancer, and colorectal cancer [35–39]. Moreover, the results from a recent study show that low expression of ELOVL6 is associated with longer survival time in HPV-positive HNSCC patients [26], which is consistent with the results obtained by the present study. There is a growing body of literature showing the importance of differential expression of serum BCHE in tumours, such as prostate cancer, pancreatic cancer, and HNSCC [40–42]. PLPP3 is mainly associated with calcific aortic valve disease, but researchers have found that low expression of PLPP3 is significantly associated with worse OS in lung adenocarcinoma and nonsmoking non-small cell lung cancer patients [43]. Consistent with this study, this suggests that PLPP3 is a protective factor. CYP4F8 can be identified as a novel therapeutic target in prostate cancer and is associated with the pharmacokinetics and toxicities of methotrexate, a fundamental drug for osteosarcoma [44, 45]. The literature also indicates that TPTE, INMT, and STAR are differentially expressed in tumours and may be associated with prognosis [46–50]. PHYH has been proven to be linked to multiple diseases, such as Refsum disease and retinitis pigmentosa, and can serve as a prognostic marker in clear cell renal cell carcinoma [51, 52]. Moreover, we showed that PHYH was a cancer-promoting gene in HNSCC, a view that is consistent with Xiong et al. [53].

Then, we analysed the literature and found that reducing cholesterol in the CD8^+^ T-cell environment enhances antitumour activity [54], and targeting the lipid metabolism of myeloid cells can reverse the immunosuppressive capacity of these cells *in vivo* and *in vitro* [55]. Therefore, we hypothesized that the immunity in tumours is linked to lipid metabolism. In this study, we found that the DEGs between the high- and low-risk groups participated in immune-related pathways by functional enrichment analysis and were related to the expression of immune cells. Moreover, in the low-risk group, increased levels of CD8^+^ T cells and CD4^+^ T cells were indicated to be involved in the antitumour immune response, consistent with previous findings [53, 56]. Furthermore, it has been shown that the ability of BCHE to hydrolyse acetylcholinesterase is involved in immune regulation [57]; aberrant expression of BCHE could promote cell proliferation and contribute to tumourigenesis [58]. The results of those two studies conducted on BCHE are consistent with the results presented here.

Finally, we utilized human oral tissue, which is common in head and neck tissue, to investigate the expression of ELOVL6 among normal oral mucosa, dysplasia, and OSCC tissues by IHC. As shown above, ELOVL6 was highly expressed in OSCC II/III, whereas no significant difference was observed in oral normal mucosa, dysplasia, or OSCC I tissue, suggesting that ELOVL6 may not be involved in OSCC development. Additionally, the clinicopathological data of the patients were collected and we found that ELOVL6 expression levels in OSCC were not correlated with their age, sex, T, N, M, vascular and perineural invasion, but only with the TNM stage. Furthermore, patients with high expression of ELOVL6 had a worse prognosis, suggesting that ELOVL6 may be a biological indicator to determine the prognosis of OSCC, which was consistent with the results of Su et al. [37]. Univariate and multivariate Cox regression analyses revealed that ELOVL6 was an independent prognostic factor in OSCC patients.

This study tried to explore the correlation between LMRGs and the prognosis of HNSCC through a risk score method. Because of the openness and operability of its data, the method is widely used in bioinformatics research and can provide new insights into the prognosis analysis of HNSCC. However, the present model still has some limitations. A single risk score cannot accurately illustrate the relevance of the gene signature to prognosis, and further validation using different cohorts is needed; that is, the results obtained with bioinformatics analysis alone are insufficient and need to be confirmed by other cohorts, such as by experimental validation [59]. Moreover, the current study did not include patients' sociodemographic feature data (body mass index, obesity, dietary factors, or lifestyles), which could influence our results.

## 5. Conclusions

In summary, we identified eight risk genes related to lipid metabolism that could predict the prognosis of patients with HNSCC and highlighted a role for ELOVL6 in OSCC by IHC. This finding may guide targeted therapy and the identification of potential biomarkers in the future. However, the sample size used in this study was relatively small, and only one prognostic target was validated.

## Figures and Tables

**Table 1:** 19 DELMRGs were identified through univariate Cox and K–M survival analyses.

Genes	K–M	Univariate	
	*P*-value	HR [95% CI]	*P*-value
*ACER1*	0.014	0.928 [0.867, 0.993]	0.030
*ACOXL*	0.020	0.870 [0.764, 0.992]	0.038
*ARSI*	0.040	1.110 [1.002, 1.229]	0.046
*ARSJ*	0.026	1.158 [1.010, 1.327]	0.035
*BCHE*	0.026	1.079 [1.009, 1.155]	0.027
*CAV1*	0.012	1.141 [1.014, 1.284]	0.028
*CYP4F8*	0.006	0.817 [0.703, 0.950]	0.009
*CYP7B1*	0.016	0.887 [0.791, 0.994]	0.039
*DPEP1*	0.014	0.907 [0.823, 0.999]	0.048
*ELOVL6*	0.001	1.299 [1.102, 1.531]	0.002
*INMT*	0.036	0.857 [0.756, 0.973]	0.017
*MOGAT2*	0.002	0.900 [0.828, 0.978]	0.013
*PHYH*	0.036	1.218 [1.002, 1.479]	0.047
*PLA2G2D*	0.015	0.923 [0.865, 0.984]	0.014
*PLPP3*	0.004	0.838 [0.722, 0.974]	0.021
*SMS*	0.023	1.496 [1.182, 1.893]	0.001
*STAR*	0.002	0.857 [0.773, 0.951]	0.004
*TPTE*	0.012	0.774 [0.616, 0.972]	0.028
*TRIB3*	0.019	1.262 [1.062, 1.501]	0.008

**Table 2:** Risk score independent prognostic analysis of HNSCC by univariate and multivariate Cox regression analyses.

	Univariate		Multivariate	
	HR [95% CI]	*P*-value	HR [95% CI]	*P*-value
Age	1.026 [1.010, 1.043]	0.002	1.018 [1.001, 1.036]	0.040
Sex	0.633 [0.438, 0.916]	0.015	0.606 [0.409, 0.898]	0.013
T	1.057 [0.875, 1.277]	0.566	1.004 [0.698, 1.442]	0.985
M	4.648 [1.461, 14.785]	0.009	5.187 [1.506, 17.865]	0.009
N	1.057 [0.872, 1.280]	0.572	1.125 [0.852, 1.486]	0.405
Stage	1.060 [0.871, 1.291]	0.560	1.011 [0.641, 1.596]	0.961
Risk score	1.893 [1.567, 2.285]	<0.001	1.945 [1.599, 2.367]	<0.001

**Table 3:** ELOVL6 expression in oral tissues.

Characteristics	*n*	ELOVL6 expression (%)		*χ* ^2^	*P*-value
		Low	High		
	138			17.802	<0.001
OSCCII/III	33	18 (54.5)	15 (45.5)		
OSCCI	38	31 (81.6)	7 (18.4)	6.036	0.014
Dysplasia	37	35 (94.6)	2 (5.4)	15.215	<0.001
Normal	30	25 (83.3)	5 (16.7)	6.010	0.014

*χ* ^2^ and *P* values are for the pairwise comparison of OSCCII/III with the other subgroups.

**Table 4:** Association between ELOVL6 expression and clinicopathological features in OSCC.

Characteristics	*n*	ELOVL6 expression (%)		*χ* ^2^	*P*-value
		Low	High		
Total	71	49 (69)	22 (31)		
Sex				
Male	50	33 (66.0)	17 (34.0)	0.718	0.397
Female	21	16 (76.2)	5 (23.8)		
Age				
<60	25	19 (76.0)	6 (24.0)	0.881	0.348
≥60	46	30 (65.2)	16 (34.8)		
Localization				
Tongue	29	19 (65.5)	10 (34.5)	2.374	0.491
Buccal mucosa	21	16 (76.2)	5 (23.8)		
Gingiva	13	10 (76.9)	3 (23.1)		
Other^a^	8	4 (50.0)	4 (50.0)		
Perineural infiltration				
Yes	20	14 (70.0)	6 (30.0)	0.013	0.910
No	51	35 (68.6)	16 (31.4)		
Vascular invasion				
Yes	7	4 (57.1)	3 (42.9)	—	0.669
No	64	45 (70.3)	19 (29.7)		
TNM				
I	38	31 (81.6)	7 (18.4)	6.036	0.014^b^
II + III	33	18 (54.5)	15 (45.5)		
Tumor size				
T1 ≤ 2 cm	12	9 (75.0)	3 (25.0)	1.805	0.649
2 cm < T2 ≤ 4 cm	36	26 (72.2)	10 (27.8)		
T3 > 4 cm	10	7 (70.0)	3 (30.0)		
T4	13	7 (53.8)	6 (46.2)		
Lymph node metastases				
N0	39	26 (66.7)	13 (33.3)	0.464	0.885
N1	19	13 (68.4)	6 (31.6)		
N2	13	10 (76.9)	3 (23.1)		
N3	0	0	0		
Distant metastases				
M0	71	49 (69.0)	22 (31.0)	—	—
M1	0	0	0		

^a^Other: carcinoma of the palate five cases and lip carcinoma three cases.

^b^ *P* < 0.05.

## Data Availability

The data used to support the findings of this study are included within the article.
